# Grazer responses to variable macroalgal resource conditions facilitate habitat structuring

**DOI:** 10.1098/rsos.171428

**Published:** 2018-01-17

**Authors:** Gavin M. Rishworth, Renzo Perissinotto, Matthew S. Bird, Noémie Pelletier

**Affiliations:** 1 DST/NRF Research Chair: Shallow Water Ecosystems, Nelson Mandela University, Port Elizabeth 6031, South Africa; 2 Department of Zoology, University of Johannesburg, Auckland Park 2006, South Africa

**Keywords:** ecosystem engineer, extant microbialite, foraging choice, stable isotope analysis, top-down pressure

## Abstract

Consumer responses to altered resource conditions can vary depending on dietary preference, resource characteristics and secondary resource features such as shelter. These can have cascading effects, especially if the consumed resource impacts on overall ecological functioning. In this study, we assessed the dietary composition of grazer communities following seasonal changes in the characteristics of their staple food-source (macroalgae). This was conducted in the living stromatolite pools growing along the coast of South Africa. Stable isotope mixing models suggested that following macroalgal bleaching in summer, metazoan consumers shifted their diet from predominantly macroalgae to a generalist composition. This has important implications for the integrity of the stromatolite matrix and its layered deposition. Where previously in winter stromatolite microalgae comprised a minor component of metazoan consumer diets, in summer, following a change in the resource conditions of macroalgae, microalgae featured more prominently in grazer diets. This seasonal grazing pressure on stromatolite-related resources probably promotes the pattern of annual layering observed in the stromatolite accretion. It also demonstrates a mechanism whereby grazer dietary shifts following a change in their preferred food resource can affect the ecosystem structure of their environment, specifically the stromatolite layering process which responds to microalgal growth or grazing conditions.

## 1. Introduction

Optimal foraging theory dictates that a consumer organism will maximize its current resource uptake up to the point at which it becomes more beneficial to switch to a new resource or resource patch [1–3]. The decision to move to a new foraging location or resource is confounded by several factors, including predator presence [4], secondary food resource attributes such as shelter [5] and the interaction between conspecifics or competitors [6]. Changes in resource availability or quality can influence the foraging behaviour of consumers. For example, ocean acidification has lowered the palatability and nutritive quality of macroalgae, which in turn invokes compensatory responses in amphipods in terms of higher overall resource intake due to lower macroalgal assimilation efficiency [7]. Alternatively, consumers might respond by selecting different, but potentially sub-optimal, food resources should the realized availability of their preferred choice deplete [8,9]. Competition for shared resources also drives foraging decisions or behaviours [10], with increased intraspecific interactions promoting dietary diversification and individual-level specialization [11]. These are important drivers of eco-evolutionary population change, whereby resource-driven competition selects for divergent foraging traits and ultimately phenotypic attributes [12].

These foraging decisions and the implications thereof are instructive in environments where foraging consequences might substantially alter ecosystem state [13]. In shallow waters, metazoan grazers can disrupt microbial mats such that the layered, sediment-stabilizing feature is not retained [14]. If this grazing (and burrowing) pressure was relaxed, these mats may be able to form sequentially layered structures [15]. From an evolutionary or geological perspective, this observation is important as microbial mats were the dominant habitat-type in shallow oceans during most of the Precambrian [16–18]. They facilitated an important role in the oxygenation of the Earth's atmosphere [19], as well as functioned as refugia for organisms seeking respite from otherwise anoxic waters [20]. Many of these mats (termed ‘microbialites’) have been preserved in the fossil record [16] because of their lithification potential, arising from the microbiologically induced/controlled deposition of calcium carbonate and the trapping or binding of sediment [21,22]. However, extant microbialites, and especially those that are layered (termed ‘stromatolites’), are rare due to several factors, including altered seawater chemistry and bioturbation disruption by metazoan grazing and burrowing activities [23].

The few known modern microbialites are confined to fringe environments that support substantial calcium carbonate concentrations [24] or features which largely exclude metazoan disruptors or eukaryotic competitors, such as hypersalinity or erosive sediment movement [25,26]. In some cases metazoans do co-occur with living microbialites without exerting an overall destructive influence [27]. These apparently unusual circumstances of coexistence are not fully understood. However, they prevail when the impact of metazoan bioturbation is less than the microbialite growth rate [24] or when selective forces act against metazoan destruction, due to the refugia benefit provided by the microbialite matrix in terms of predator avoidance, ambient buffering or oxygen supply [27]. Recent evidence suggests that metazoan–microbialite coexistence might also be facilitated when there is an alternative food source for grazers, rather than the microbialite microalgae themselves [28]. This restricts the grazing impact on the microbialite matrix, therefore, enabling unimpeded layering, and provides grazer control on other primary producers (macroalgae particularly) that might otherwise outcompete the microbialite microalgae [29].

**Figure 1.**
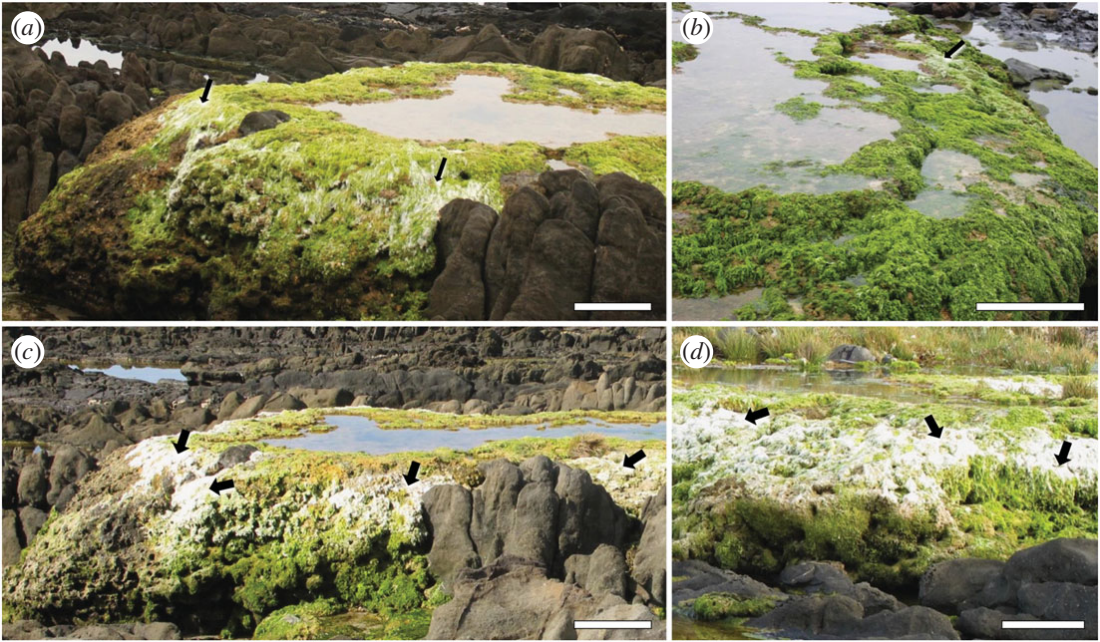
Peritidal stromatolite barrage pools during winter (*a*,*b*) and summer (*c*,*d*) at Seaview and Schoenmakerskop, South Africa, showing the state of pool macroalgae (*Ulva* spp.), with arrows indicating bleached areas. Photographs were taken in winter (August 2015: [28]) and summer (January 2016: this study) by Ross-Lynne Weston and Lynette Clennell, respectively. Scale bars (0.5 m) are reflective of foreground objects.

Avoidance of microbialite microalgae by metazoans as a reason for microbialite persistence assumes preferential selection of macroalgae. However, field observations suggest that the seasonal variability of salinity and temperature at peritidal stromatolite pools along the South African coastline [30] creates cyclical dynamics in macroalgal biomass [31]. Bleached, degenerated conditions of *Ulva* spp. predominate during summer periods (as a response to increased irradiance, temperature and desiccation: [32,33]) when there is a more persistent freshwater pool state (figure 1). This suggests a degree of variability in macroalgal resources available to stromatolite-inhabiting metazoans. In this study, we sought to capitalize on this seasonal ‘natural experiment’ by assessing the foraging responses of metazoans to changing macroalgal resource conditions. Stable isotopes were used to this end as they are a useful overall indicator of consumed resources and dietary preferences [34]. Observation of diet-switching following resource condition change requires a synergistic stable isotope signature, as well as an understanding of individual-level physiological growth responses in terms of isotopic trophic fractionation [35]. This former premise was met for the metazoans investigated in this study, which have a rapid dietary assimilation and tissue turnover rate (5–10 days: [36]). We predicted that metazoan dietary signatures under bleached macroalgal resource conditions would reflect a greater foraging reliance on other non-macroalgal resources (generalist diet), while non-stromatolite items would remain the predominant overall dietary contributor given the consistently observed metazoan–stromatolite coexistence at these sites [27]. This is the first study to contextualize consumer responses to shifting resource variability in a microbialite ecosystem.

## 2. Material and methods

### 2.1. Study site

Stromatolites forming along the South African coastline are restricted to the peritidal zone, where freshwater from subterranean seeps meets periodic marine incursion during storm or tidal surges [30]. At this salinity interface zone, optimal nutrient conditions in terms of nitrogen from the seeps and phosphorus from the ocean create suitable conditions for benthic-dominated stromatolite biomass [30,37]. The stromatolites, which are principally comprised of cyanobacteria and diatoms [38], accrete at a maximum rate of 2–5 mm per year [39] forming barrage pools (figure 1) up to 1 m in depth, where the bulk of the stromatolite biomass is contained.

This study was conducted within the main barrage pools of three sites along the southern South African coastline: Cape Recife (site A; 34°02′42.13′′ S, 25°34′07.50′′ E), Schoenmakerskop (site B; 34°02′28.23′′ S, 25°32′18.60′′ E) and Seaview (site C; 34°01′03.16′′ S, 25°21′56.48′′ E). These same pools were surveyed during a previous winter season [28], when macroalgal biomass appeared healthy (figure 1*a,b*).

### 2.2. Data collection

Samples were collected from barrage pools at all three sites, in August 2015 (these data are also presented in Rishworth *et al.* [28]) and February 2016 during the austral winter and summer, respectively. Physico-chemical measurements were recorded in each pool using a YSI 6600-V2 multi-parameter probe (YSI, Yellow Springs, USA): temperature (°C), salinity, oxygen (mg l^−1^), turbidity and pH. Nutrient conditions in terms of dissolved inorganic nitrogen (DIN) and phosphorus (DIP) from the seeps, pools and ocean were assessed using spectrophotometric methods (see [30]). Phytoplankton and benthic microalgal biomass were also assessed, using acetone-extracted chlorophyll-*a* as a proxy, as recorded on a Turner 10-AU fluorometer (Turner Designs, Sunnyvale, USA). As the formative component of the stromatolite environment, community class composition of the benthic microalgae was also assessed using a BenthoTorch (bbe Moldaenke, GmbH, Schwentinental, Germany). Further specific details on these methods are presented elsewhere [37,38].

Particulate organic matter (POM), which included the phytoplankton, was isolated from the seeps, pools and ocean by filtering approximately 1 l of water onto pre-combusted (6 h, 450°C) glass-fibre filters (GF/F; 0.7 µM). Sediment organic matter (SOM) was collected from benthic grabs at the three barrage pools, whereafter all noticeable shell, plant or animal material was discarded. Stromatolite SOM was also collected, this being sampled from cores within the stromatolite accretion, after removing the upper 2 cm which contains the actively accreting microalgae [38]. Detrital material (decaying plant matter) was collected from all seeps. When present, above-ground, living biomass of macrophytes (forbes or dicotyledons and grasses or monocotyledons) and macroalgae was hand-collected from the seeps, pools and ocean. Microalgae were collected from rock scrapes within the pools (non-stromatolite forming) as well as directly from the stromatolite accretion, these being scrapes of the surface 1–2 mm layer.

Primary consumers were collected from the main barrage pools using a combination of cores, rock scrapes and sweep netting. Stromatolite infauna were differentiated as those directly inhabiting the stromatolite matrix, collected using a 1.7 cm diameter stainless steel corer [29], with the remainder classified as epifauna. Secondary consumers (fish, crabs, shrimps) were hand-collected using 1 mm nets.

Sampling effort at each site for all community components, when present, was continued until sufficient material was collected to provide at least 3–5 replicate stable isotope measurements for each taxon or organic matter source per site for both winter (August 2015: [28]) and summer (February 2016) surveys.

### 2.3. Stable isotope sample processing

All community components were frozen directly (−40°C) before being dried (60°C, ≥48 h). Both filters containing POM as well as SOM were dried directly, after removing potential contaminants. Detrital material, macrophytes, macroalgae and microalgae were inspected for epibiont or other contaminants (which were subsequently discarded) before being dried. Infauna and epifauna were examined under a dissecting microscope and sorted according to lowest, appropriate taxonomic level following Rishworth *et al.* [28] before drying. After complete desiccation, all samples were ground to a fine, homogenized powder using an agate pestle and mortar.

The presence of inorganic carbonates (shells, chitinous exoskeletons, bones) associated with organic material can skew the meaningful isotopic *δ*^13^C signature, because only the organic component is assimilated between trophic levels [40,41]. As such, inorganic carbonates should be removed mechanically or chemically prior to spectrometric analysis. In addition to the removal of obvious shell fragments prior to drying, crushed samples were exposed to acid treatment following Jacob *et al.* [40], as elaborated upon in Rishworth *et al.* [28]. Prior to this, approximately half of all samples were separated as non-acidified replicates, to avoid unwanted acidification effects on the *δ*^15^N signature [41,42]. The ‘acidified’ *δ*^13^C signatures for samples that had insufficient material for both an acidified and non-acidified replicate were calculated using linear regression relationships (see electronic supplementary material, figure S1) of known sample-specific acidification effects (for further explanation see [28]). Sediment, microalgae and macroalgae samples were treated dropwise with 1 N HCl until effervescence of volatilized CO_2_ from the inorganic carbonates had ceased. Distilled water subsequently was added to remove hygroscopic crystals formed because of the high inorganic carbonate content of these samples, which would otherwise interfere during grinding and damage spectrometric equipment if not removed [43]. Rinsing followed by centrifugation (2000*g*) was repeated three times through the addition of distilled water (3× sample volume), carefully removing the liquid supernatant between each subsequent spin. Macroinvertebrate material and POM filters were treated dropwise with 0.25 N HCl, but not rinsed afterwards. Prior to the addition of HCl to macroinvertebrate samples, lipids were extracted (following [44]) as these can also skew the accuracy of *δ*^13^C [42]. All samples again were dried and homogenized by crushing and grinding.

Sediment and filter samples were stored in sterilized 2 ml polypropylene vials and aluminium foil, respectively, prior to analysis. Macrophyte, detritus, macroalgae and microalgae powder was carefully weighed into sterilized tin capsules (5 × 9 mm; Säntis Analytical AG, Switzerland) and sub-sampled into 3–5 replicates (1.0 ± 0.05 mg each). Macroinvertebrate samples were similarly weighed into 3–5 sub-sampled replicates (0.5 ± 0.05 mg each) unless there was insufficient material, whereby samples from several specimens across the same species or taxa were combined.

Isotopic analyses were conducted at iThemba Laboratories (Johannesburg, South Africa) using a Flash HT Plus elemental analyser which was connected to a Delta V Advantage isotope ratio mass spectrometer through a ConFloIV interface (equipment supplied by ThermoFisher, Bremen, Germany). This provided the relative carbon (^13^C and ^12^C) and nitrogen (^15^N and ^14^N) ratios. Following convention, SI data were presented as the fractional difference between samples and known international standard values (‰), these being Pee Dee Belemite carbonate (*δ*^13^C) and atmospheric N_2_ (*δ*^15^N):

$$\delta X=\frac{\left(R_{\mathrm{sample}}/R_{\mathrm{standard}}\right)}{R_{\mathrm{standard}}}\times 10^{3},$$

where *X* is ^13^C or ^15^N and *R* is the ^13^C : ^12^C or ^15^N : ^14^N ratio. Between every 24–25 samples, a known standard sample was run to correct spectrometric SI values against, these being Merck Gel or Urea (IVA Analysentechnik e.K., Meerbusch, Germany). The 1*σ* precision between standards (*n* = 95) was ±0.17 and ±0.12‰ for *δ*^13^C and *δ*^15^N, respectively.

### 2.4. Analysis

All data were analysed in R [45] using the ‘nlme’, ‘SIBER’, ‘MixSIAR’ and ‘mvabund’ packages [46–49]. Isotopic niche space provides a quantitative metric of expanse per trophic guild, an informative tool for defining the variability of different food webs and the broadness or complexity of organic matter transfer between trophic levels [47,50]. In this study, these spatial metrics were quantified using Bayesian ellipses, specifically the uncorrected and corrected forms of the standard ellipse area (SEA and SEAc), the latter of which is robust for small sample sizes [47].

Stable isotope analysis allows for the mathematical determination of the dietary composition of consumer organisms if the stable isotope signatures of all consumed resources are known, following the principles of dietary assimilation [34,51]. Recent developments in these procedures have enabled biases such as differential fractionation during assimilation or variability associated with resource elemental composition, availability or partitioning, to be mathematically overcome in ‘mixing models’ [48,51]. As such, the dietary composition of all dominant consumer groups associated with the stromatolite barrage pools [28] could be reasonably determined. Resource components (inlet detritus, inlet POM, inlet SOM, ocean macroalgae, ocean POM, pool microalgae, pool POM, pool SOM, pool macroalgae, stromatolite microalgae and stromatolite SOM) were selected based on known species' feeding ecology from previous assessments made through site-specific observations of resource conditions [28,29,31]. The SI signatures of all resources were compared using a linear mixed-effects approach under the generalized least-squares framework [28]. This was done by first determining the random structure (sampling site and organic matter source) that optimized residual variability, and then testing the significance of sampling sites and organic matter sources as fixed effects [52,53]. All resources that were statistically indistinguishable were combined *a priori* [54]. A conservative estimate of dietary fractionation was incorporated because of the ability of ‘MixSIAR’ to incorporate this uncertainty within a residual error term [55]. This was set as 1.0 ± 0.25 and 2.0 ± 0.5‰ for *δ*^13^C and *δ*^15^N, respectively, based on published accounts [56] and site-specific trophic-level data (this study; [28]). Dietary composition was then determined for each primary consumer guild taxa using a mixing model and expressed per taxonomic group and study site, both of which were nested as random effects [57]. Dietary proportions of dominant taxa were compared between systems of varying macroalgal states (bleached: this study; non-bleached: [28]) using a multivariate generalized linear modelling (GLM) approach [49], accounting for site, location (infauna or epifauna), season and taxonomy as possible explanatory variables. This multivariate method accounts for the mean–variance relationship between univariate datasets (i.e. each taxon's diet) in a more accurate manner than conventional distance-based multivariate approaches [58]. The multivariate GLM was fitted using the ‘mvabund’ package and the explained deviance (D) of predictor variables compared using a multivariate ANOVA (‘anova’, p.uni = ‘adjusted’, nBoot = 10 000; [49]). All model assumptions were tested and met in terms of normality and homogeneity of residuals [49,53].

Results presented are largely those from summer data as the winter data are published in Rishworth *et al.* [28]. However, winter stable isotope data are re-analysed in this study for comparison to the summer data.

Results are expressed as mean ± s.d. (unless indicated otherwise) and an *a priori* significance level of *α* = 0.05 was set.

## 3. Results

### 3.1. Study site features

In both seasons, depth stratification was evident at all three sampling locations, especially for salinity where surface waters were virtually fresh (less than or equal to approx. 5) and bottom waters marine (greater than 30; electronic supplementary material, table S1 and table 1 in Rishworth *et al.* [28]). Higher concentrations of DIN were derived from inlet waters, decreasing from there to middle pools and the ocean, with site A reflecting the lowest concentrations overall. Across most sites the ocean had marginally higher DIP concentrations, apart from at site C where a high inlet source was apparent. Reflecting overall nutrient conditions, both pelagic and benthic chlorophyll-*a* biomass were usually highest at site C, with cyanobacteria dominating the stromatolite microalgal community in summer (greater than 80%), but co-occurring equally with diatoms in winter. The most noticeable seasonal difference was for pool temperature, which was warmer in summer (approx. 21.1–24.1°C; electronic supplementary material, table S1) compared to winter (14.9–18.8°C; table 1 in Rishworth *et al.* [28]). Although a year-round tidal occurrence [30], more-recent ocean overtopping had occurred prior to the summer sampling event, reflecting higher salinity and turbidity in bottom waters.

**Table 1.** Multivariate generalized linear model of primary consumer dietary contributions in relation to season (‘winter’ versus ‘summer’), location relative to the stromatolite matrix (‘infauna’ versus ‘epifauna’) as well as the interaction of season with each primary consumer species (see figure 3). The proportional deviance (*D*%) and the test significance (*sensu* [49]) of the overall multivariate model, as well as for each univariate model of dietary source nested within the overall model, are shown. The *D*% is differentiated according to that explaining the dietary variability (Di.) and the predictor variability (Pr.). Positive or negative coefficient (C) effects of predictors are indicated by directional arrows. d.f., degrees of freedom; OM, organic matter; SOM, sediment OM; POM, particulate OM.

predictor (Pr.):	season^winter^					location^infauna^					season : species			
	*D%*					*D%*					*D%*			
dietary contribution (Di.):	Di.	Pr.	d.f.	*C*	*p*	Di.	Pr.	d.f.	*C*	*p*	Di.	Pr.	d.f.	*p*
overall	44	—	1	—	***	4	—	1	—	0.2	52	—	10	**
inlet														
detritus	67	18		^↑^	***	0	1		^↑^	0.8	32	7		0.4
OM	0	0		^↓^	0.8	6	13		^↑^	0.6	94	17		*
Pool														
Malacostracans	4	1		^↓^	0.5	5	8		^↓^	0.7	91	11		0.2
macroalgae	62	19		^↑^	***	5	16		^↓^	0.5	33	8		0.4
microalgae	40	12		^↓^	***	1	3		^↓^	0.8	59	15		°
OM	65	13		^↓^	***	2	5		^↓^	0.8	32	5		0.4
stromatolite														
microalgae	29	6		^↓^	**	8	18		^↑^	0.5	63	11		0.2
SOM	30	7		^↓^	**	3	7		^↑^	0.8	67	14		°
ocean														
macroalgae	14	1		^↑^	0.3	3	3		^↑^	0.8	83	7		0.4
POM	74	23		^↓^	***	8	26		^↑^	0.3	18	5		0.4

Test significance:

***p <* *0.001.

**p <* *0.01.

*p <* *0.05.

°*p *<* *0.10.

### 3.2. Trophic composition

The stromatolite pool trophic community was broadly grouped according to organic matter sources (terrestrial/inlet as well as ocean material as allochthonous sources; pool material as autochthonous sources), primary consumers (amphipods, isopods, tanaids, chironomids, polychaetes, oligochaetes and gastropods; see electronic supplementary material, table S2 for site-specific samples collected and their relative abundance) and secondary consumers (fishes, crabs and shrimps), which largely demonstrated clear separation according to trophic guild (electronic supplementary material, figure S2 and figure 1 in Rishworth *et al.* [28]). Only the polychaete *Composetia* cf. *keiskama* overlapped consistently between the primary and secondary consumer guilds in terms of *δ*^15^N signatures in summer (electronic supplementary material, figure S2). Site-specific trends were apparent whereby, for example, the primary consumer guild was enriched in terms of nitrogen isotopes from Cape Recife, to Schoenmakerskop and to Seaview (*δ*^15^N = 7.1 ± 1.2, 7.7 ± 1.0, and 8.0 ± 1.4, respectively, in summer). This site-specific trend also reflected for carbon isotopes of the organic matter sources (electronic supplementary material, table S3 and table 3 in Rishworth *et al.* [28]).

**Figure 2.**
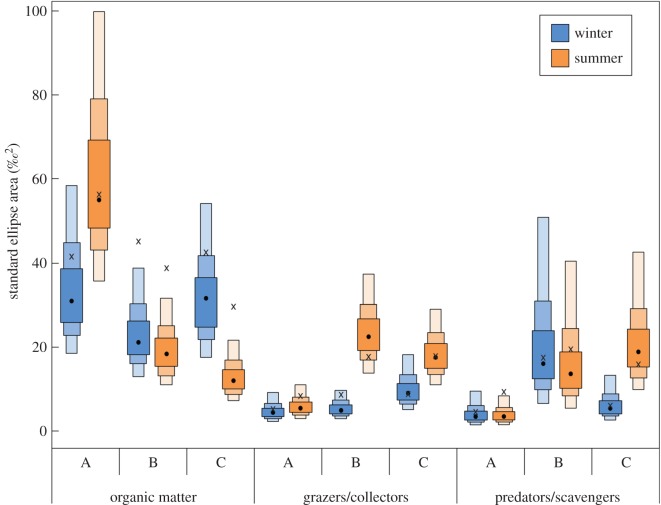
Niche dimensions in terms of the standard ellipse area (SEA) of trophic guilds sampled at three stromatolite locations (A: Cape Recife; B: Schoenmakerskop; C: Seaview) during winter and summer. SEA values are portrayed as their mean (black dot), bordered by the 50%, 75% and 95% quantile boxes, as well as the corrected SEA (SEAc; ‘×’ symbols) which accounts for small sample sizes.

The categorization of trophic niche areas demonstrated differences between sites, with Cape Recife reflecting the most and Seaview the least variability in organic matter sources (figure 2). By contrast, both the primary and secondary consumer guilds were broader at sites B and C compared to site A (figure 2). These trends also differed from the trophic niche width observed during the winter months within the stromatolite pools, where the niche width was constricted, especially for the grazer and detritivore community (figure 2). Barring this, all other seasonal comparisons in terms of niche width were similar, especially when comparing SEAc rather than SEA values, with the only exception being the expanded predator/scavenger guild width at site C during summer (figure 2). This was probably due to the presence of the crab *Varuna litterata* which was absent in winter (electronic supplementary material, figure S2).

**Figure 3.**
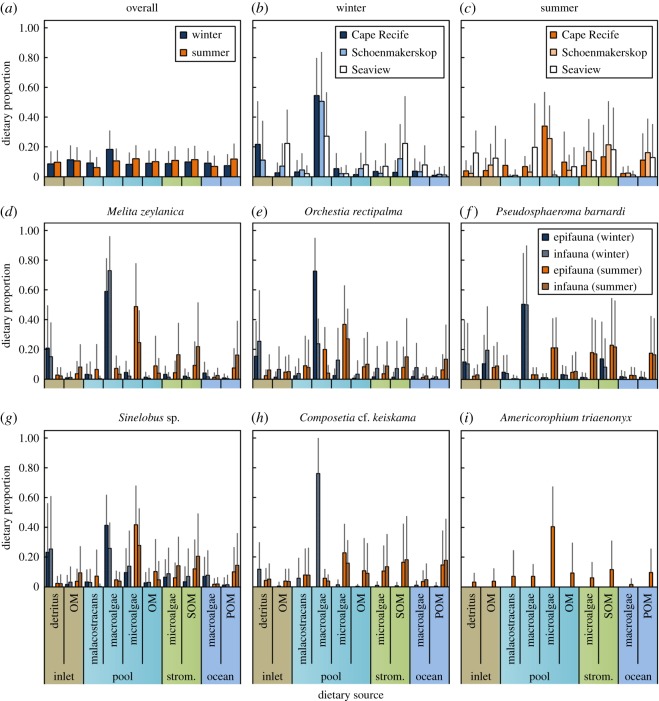
Dietary proportions (±s.d.) of metazoan primary consumers within living stromatolite pool at three sites along the South African coastline. Panels are differentiated by the overall seasonal dietary contributions (*a*), the site-specific seasonal contributions (*b*,*c*) and the dietary proportions of each dominant macrofauna species (*d*–*i*), separated according to season and location relative to the stromatolite matrix (within, ‘infauna’ or outside, ‘epifauna’).

As in winter (table 3 in Rishworth *et al.* [28]), there was a clear distinction between stromatolite microalgae and other organic matter sources in terms of *δ*^13^C signatures in summer (all *p *< 0.05), with the only exceptions being ocean POM, pool SOM and inlet detritus (electronic supplementary material, table S3). These latter sources that had similar carbon isotope signatures were easily distinguished from the stromatolite material in terms of *δ*^15^N (electronic supplementary material, figure S2). Therefore, a clear separation of organic matter sources with regards to the dietary mixing model could be generated (figure 3). Both inlet and pool SOM and POM were combined *a priori* for consideration in the mixing model as these were ecologically indistinguishable in terms of their isotope signatures (electronic supplementary material, figure S2 and table S3).

### 3.3. Consumer diets

The higher apparent trophic position of *C.* cf. *keiskama* (Polychaeta, Nereididae) compared to other primary consumers (electronic supplementary material, figure S2), as well as the known omnivorous diet of these burrowing worms [59], prompted the inclusion of malacostracans (Crustacea; in this study being amphipods, isopods and tanaids) as a further dietary source, in addition to the organic matter sources discussed above (electronic supplementary material, table S3). This inclusion, together with that of inlet detritus, differed from the results presented in Rishworth *et al.* [28]. Despite the consideration of inlet detritus and malacostracans in winter primary consumer diets, overall estimates (see ‘Winter’ bars in figure 3) remained largely unchanged from those published in Rishworth *et al.* [28]. There was less than or equal to 20% contribution of inlet detritus to all consumer diets, but virtually no malacostracan signatures. This suggests that dietary misidentification, while certainly an important source of error in stable isotope mixing models [51], had minimal impact on the results or interpretations presented in Rishworth *et al.* [28]. Nonetheless, the results presented in this study are more accurate and similarly suggest that pool macroalgae remained the overwhelmingly dominant dietary resource in the winter food web (figure 3*b*), with little to no stromatolite material consumed.

In contrast, and also reflecting the broader observed trophic niche widths discussed previously (figure 2), summer primary consumer diets reflected a significantly different and more-generalist pattern (*p *< 0.001, table 1; figure 3*c*). During summer conditions when macroalgae associated with stromatolite pools appeared bleached (figure 1*c*,*d*), instead of relying on pool macroalgae, the dominant dietary resource consumed was pool microalgae, although this trend differed between sites (figure 3*c*) and species (figure 3*d–i*). Summer diets suggested a higher reliance on stromatolite-related material (microalgae and SOM), especially for species such as *Pseudosphaeroma barnardi* (Isopoda; figure 3*f*) and infauna compared to epifauna (table 1). Infauna generally consumed more stromatolite material (combined deviance explained: 25%, *p *> 0.5) and particulate or sediment OM (39% D combined) from allochthonous sources compared to epifauna (table 1). Species such as *Melita zeylanica* (Amphipoda; figure 3*d*) and *C.* cf. *keiskama* (Polychaeta; figure 3*h*), that almost exclusively relied on pool macroalgae during winter, shifted their diets to predominantly pool microalgae and stromatolite-related material in summer. According to sampling locations, Seaview (site C) consumer diets were the most similar between seasons (figure 3*c*), with there still being a more-generalist dietary composition in summer compared to winter.

Key overall differences in seasonal diets were: a higher reliance on detritus (resource-specific deviance explained: 67% D, *p *< 0.001) and pool macroalgae (62% D, *p *< 0.001) during winter, shifting to a broader resource niche that included pool microalgae (40% D, *p *< 0.001) and OM (65% D, *p *< 0.001), stromatolite microalgae (29% D, *p *< 0.01) and SOM (30% D, *p *< 0.01), as well as ocean POM (74% D, *p *< 0.001) during summer macroalgae-bleached conditions (table 1). Malacostracans were not an important resource for consumers, although some species (e.g. *C.* cf. *keiskama*) portrayed an omnivorous trophic guild (electronic supplementary material, figure S2, figure 3).

## 4. Discussion

Under altered conditions of resource availability or quality, consumers respond either by increasing the quantity of their preferred resource despite its reduced quality [7], by changing their dietary preference [9], or by relocating to habitats where resources might be more abundant. This study investigated the dietary response by macrofaunal consumers associated with living stromatolite pools following a seasonal shift in macroalgal conditions (figure 1), this being the resource that forms their dominant dietary component [28]. Instructively, invertebrate grazers and detritivores altered their dietary niche, shifting away from an almost exclusively macroalgae-comprised diet in winter [28] to a generalist diet in summer (figure 3).

The most likely driver of this shift is the decrease in overall biomass and nutritive quality of bleached macroalgae in summer (figure 1*c*,*d*) compared to winter. Macroalgal bleaching is a physiological mechanism whereby the algal thallus loses photosynthetic pigment due to stress associated with high irradiance or temperature [32]. Although bleaching is not always lethal, and can indeed be a facultative adaptation towards screening the lower algal biomass against harmful UV radiation [60], it does invoke variable responses with regards to thallus nutritive quality and palatability. In some macroalgae, such as kelps and rhodophytes, bleached sections are favoured by grazing invertebrates (e.g. [61]) because they have probably lost defensive secondary metabolites [62]. For example, grazing amphipods and isopods that feed on seaweed wrack that has washed ashore through wave action, prefer aged and degraded material as it is more palatable [63]. However, the dominant macroalgae associated with the stromatolite pools, *Ulva* spp., is a palatable seaweed for most grazers, often favoured in choice experiments over other macroalgae [64,65]. Additionally, under stressful conditions (such as high temperatures and irradiance), nutritive qualities of bleached *Ulva* diminish significantly [66]. For these reasons, as well as the reduction in macroalgal biomass following bleaching or high irradiance (*sensu* [33]), summer conditions of the dominant macroalgae at the stromatolite pools represent restricted resource availability for grazers. Hence, our *a priori* prediction that macroalgae comprises a lower proportion in consumer diets in summer compared to winter is supported.

Macrofaunal density within the stromatolite matrix demonstrates little variability despite seasonal shifts in environmental conditions [29,30]. Although there is evidence that macroalgal biomass (using percentage cover as a proxy) is inversely correlated with macrofaunal abundance [29], seasonal trends suggest that fluctuating resource conditions overall result in dietary diversification, as this study demonstrates, rather than population-level density responses. Seasonal changes, especially those associated with temperature, will largely determine the physiological requirements of the macrofaunal consumers. Being ectotherms, warmer temperatures in summer [30], especially for those invertebrate consumers that are mobile or active, would increase their metabolic rate [67]. This requires a higher energetic input to sustain metabolism. The dietary analysis presented in this study suggests that grazers rely on a broader resource base to meet their summer energetic requirements: macroalgae alone, especially in a bleached state, therefore, does not appear sufficient in this regard. Recent syntheses have informatively shown how consumers experiencing stressful or growth-limiting conditions can reflect broadened isotopic niche widths because of physiological variability in trophic fractionation [35]. In turn, this would be interpreted as dietary diversification under stable isotope trophic premises. Although the metazoan consumers sampled in this study experience minimal seasonal stress-related pressures, assuming that stable population densities are an accurate proxy for this [29], growth-related individual specialization within metazoan populations during warmer summer temperatures, for example, might contribute towards some of the interpreted summer dietary generalization (*sensu* [35]). The current study accounted for this possible uncertainty through the conservative trophic fractionation estimate [55]; however, future research should quantify possible stress-induced physiological responses in populations associated with these habitats to precisely determine its influence. The metazoans sampled in this study have a high tissue turnover rate (5–10 days: [36]) and consequently stresses occurring concomitantly with resource fluctuations, if any were apparent, might express as intra-seasonal variability rather than accounting for the differences between the two seasonal dietary observations.

From the perspective of the ecosystem-engineering stromatolite microalgae, these results are revealing. Stromatolite layering forms as a result of the cyclical process of pioneer and climax microalgae shifts following seasonal or stochastic processes [22]. For example, the marine stromatolites of the Bahamas, where seasonal temperature variability is minimal, respond to ephemeral processes of sediment burial and re-emergence [26], whereas the layered precipitation of those in Shark Bay, Australia, correlates with seasonal patterns of salinity and carbonate-rich groundwater supply entering the hypersaline coastal embayment [25]. Previous work on the South African stromatolites has suggested that the layering process might be annual [39] although the mechanism of this could not be verified. Subsequently, Rishworth *et al.* [30] demonstrated seasonal variability in winter- and storm-associated salinity patterns, which are a driver of microalgal community and biomass patterns [38]. This provided a possible explanation for the seasonally driven stromatolite layering. However, the results from this study provide further insight. It is likely that the sequential layering is indeed annual, whereby, in addition to the salinity-driven shifts in the microbial community, the dichotomous seasonal pressures exerted by metazoan grazers might enhance this. Lack of metazoan grazing on stromatolite microalgae during winter would promote, or at least not inhibit, stromatolite growth [28], whereas the greater reliance on stromatolite-related material during bleached macroalgae conditions (this study) would restrict stromatolite microalgal/microbial growth. This is especially apparent where infaunal consumers living directly within the stromatolite matrix consumed more stromatolite material than those living as epifauna (table 1), unlike what was observed when macroalgae dominated consumer diets [28]. Grazer responses to variable macroalgal resource conditions, therefore, contribute towards facilitating the seasonal layering of South African peritidal stromatolites. These observations made from the dietary mixing model results are enhanced by the clear distinction in isotopic source signatures (electronic supplementary material, table S3), probably as a result of distinct photosynthetic carbon sequestration pathways between primary producer groups from the marine, stromatolite and terrestrial environments (e.g. [68]).

Confounding these responses is the site-specific variability associated with stromatolite-forming locations (electronic supplementary material, table S1). A trend of increased nutrient load from Cape Recife to Seaview is well supported [30]. This is reflected in the stromatolite trophic community, whereby sites receiving higher quantities of anthropogenic DIN reflect enriched *δ*^15^N [28], a clear indication of human-driven pollution [69], and which was also observed in the consumer *δ*^15^N signatures in this study (electronic supplementary material, figure S2). This enrichment might affect the dietary fractionation or preferential routing of enriched compounds, a feature that was partly accommodated for by incorporating conservative standard errors in trophic fractionation scores and sampling sites as random variables in the mixing models [51,56]. Nonetheless, future studies should quantify the direct effect of *δ*^15^N enrichment, as this might introduce some uncertainty to diet estimates. Nitrogen enrichment is also known to increase the resilience of *Ulva* sp. following the effects of bleaching [70]. Consequently, site C (Seaview), where nitrogen input was the greatest, reflected the largest proportion of macroalgae within the metazoan consumer diets and also overall demonstrated the smallest difference between summer and winter diets, although macroalgal consumption similarly to the other sites was less in summer (figure 3*c*). This suggests that the effect of nutrient buffering bolstered the resource condition of macroalgae at Seaview and enabled the metazoan primary consumers to minimize their summer consumption of microalgae overall. In stromatolite environments, microalgae are generally considered to have a high degree of resilience to stressors such as temperature and salinity (e.g. [25,26]); this resource, therefore, might be in better condition for grazing consumers in summer. The shift in dominant dietary components from macroalgae in winter to microalgae in summer is, therefore, expected. Additionally, Schoenmakerskop (site B) experiences substantially warmer main pool conditions (approx. 2–4°C warmer) than the other sites [30], which explains why macroalgae reflected the lowest dietary proportions at this location (figure 3*c*) and also the high reliance on stromatolite-related and other microalgal sources. The peritidal stromatolite ecosystems reflect an interesting case of coevolution and coexistence between macrofauna, macroalgae and the stromatolite-forming microbes (microalgae and cyanobacteria). The heightened anthropogenic loading at the Seaview location, and consequent expected future increase in coastal eutrophication in general [30], would possibly impact on stromatolite layering if the nitrogen-bolstered macroalgal community shifts to being a year-round grazer resource. Long-term monitoring at a heavily impacted site could elucidate how this might affect stromatolite layering.

In conclusion, this study highlights the role of top-down grazing pressure towards controlling the ecological–engineering process of stromatolite laminar formation. However, seasonal cycles of microalgal consumption of microbial mats are not solely sufficient to enable stromatolite layering [14,15]. Other factors need to also be in place, such as elevated water calcium carbonate saturation [21] and predominant exclusion of bioturbating organisms or their effects [25,27]. Nonetheless, this is the first study to demonstrate how, in modern and actively accreting microbialite mats, grazer pressures promote sequential layering if these effects are ephemeral or seasonal, rather than destroy or homogenize the mat. The mechanism of this appears to be the change in resource conditions of macroalgae, which, if maintained in an optimal state of nutrition and availability, would probably otherwise be preferred by grazing metazoans over the stromatolite and other microalgae. Modern stromatolites thrive under a balance of forces which together promote their persistence at rare localities, especially those where grazing pressures remain [24–26,28,29]. Future work at the peritidal stromatolites should investigate the role of grazer exclusions, algal-choice feeding experiments, stress-induced isotopic fractionation [35] and *δ*^15^N enrichment effects, and how top-down predatory interactions [71] dictate foraging choices by grazers, especially considering secondary features of dietary items such as refugia protection. This is the first study to assess grazer responses following seasonal resource variability in a stromatolite habitat. Although the pattern of consumer dietary diversification following resource depletion has been demonstrated in many ecosystems (e.g. [72]), a knowledge gap exists on how these effects might translate to other microbialite habitats or environments where consumers are similarly potential ecosystem engineers (*sensu* [13]).

## Supplementary Material

All supplementary tables, figures and data

## Data accessibility

All data used in this manuscript are presented in electronic supplementary material, table S4 or available in the Open Access manuscript, Rishworth *et al.* [28].
