# Supplementary material for: Grazer responses to variable macroalgal resource conditions facilitate habitat structuring
Source: R Soc Open Sci. 2018 Jan 17;5(1):171428. doi: 10.1098/rsos.171428 (PMC5792922; doi:10.1098/rsos.171428)
Supplement: All supplementary tables, figures and data [file rsos171428supp1.pdf]

Grazer responses to variable macroalgal resource conditions facilitate habitat structuring

Gavin M. Rishworth[1,*], Renzo Perissinotto[1], Matthew S. Bird[1,2], Noémie Pelletier[1]

[1]DST/NRF Research Chair: Shallow Water Ecosystems, Nelson Mandela University, Port Elizabeth 6031, South Africa

[2]Department of Zoology, University of Johannesburg, Auckland Park 2006, South Africa

*Corresponding author: gavin.rishworth@gmail.com

Supplementary material

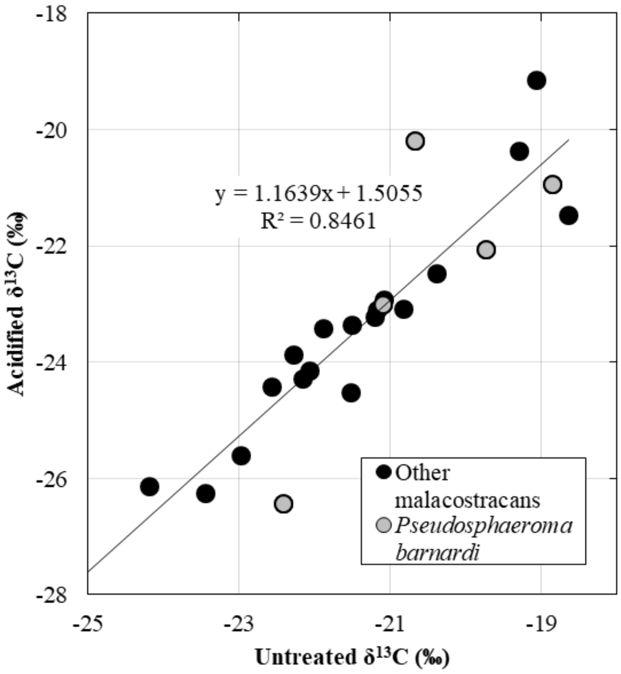

Figure S1. Linear relationship between dried and defatted malacostracan samples that had been treated with 0.25N HCl compared to samples that were untreated. This well-fitted relationship (R^2 = 0.85) was used to calculate the acidified $\delta^{13}C$ signature for samples for which there was insufficient material to quantify both an acidified and untreated estimate (see ‘Materials and methods’ and Rishworth et al. [28]).

Table S1. Physico-chemical and primary producer (phytoplankton and benthic microalgae) biomass associated with the three stromatolite study sites (A: Cape Recife; B: Schoenmakerskop; C: Seaview) along the South African coastline from which stable isotope collections were made during February 2016 (winter data are presented in Rishworth *et al.* [28]). Data are reflective of main, barrage pool locations, unless otherwise indicated.

Summer		**Temp (°C)**	**Sal**	**Turb (NTU)**	**DO (mg.l^{-1})**	**pH**	**DIN (µM)** I; P; O	**DIP (µM)** I; P; O	**Pelagic chl-*a* (mg.m^{-3})**	**Benthic chl-*a* (mg.m^{-2})** Cy;Ch;Di
A	Surface	23.1	1.7	0	11.0	8.4	57; 31; 2	0.3; 0.2; 0.4	1.2 ±0.0	368.1 ±38.3
	Bottom	21.1	32.7	8.3	12.0	8.5				90%; 3%; 8%
B	Surface	24.2	6.2	0	8.2	8.3	313; 67; 5	0.4; 0.4; 0.7	1.2 ±0.1	393.2 ±159.8
	Bottom	23.9	32.4	6.7	6.5	8.3				85%; 11%; 4%
C	Surface	21.1	4.1	0	5.7	8.0	396; 110; 4	1.2; 0.7; 0.9	2.8 ±0.1	1145.5 ±342.7
	Bottom	21.3	30.7	1.4	7.1	8.2				80%; 9%; 12%

Temp (*Temperature*); Sal (*Salinity*); Turb (*Turbidity*); DO (*Dissolved Oxygen*); DIN (*Dissolved Inorganic Nitrogen*); DIP (*Dissolved Inorganic Phosphorus*); I (*Inlet*); P (*Pool*); O (*Ocean*); Cy (*Cyanophyta*); Ch (*Chlorophyta*); Di (*Bacillariophyta*, mostly *Diatoms*)

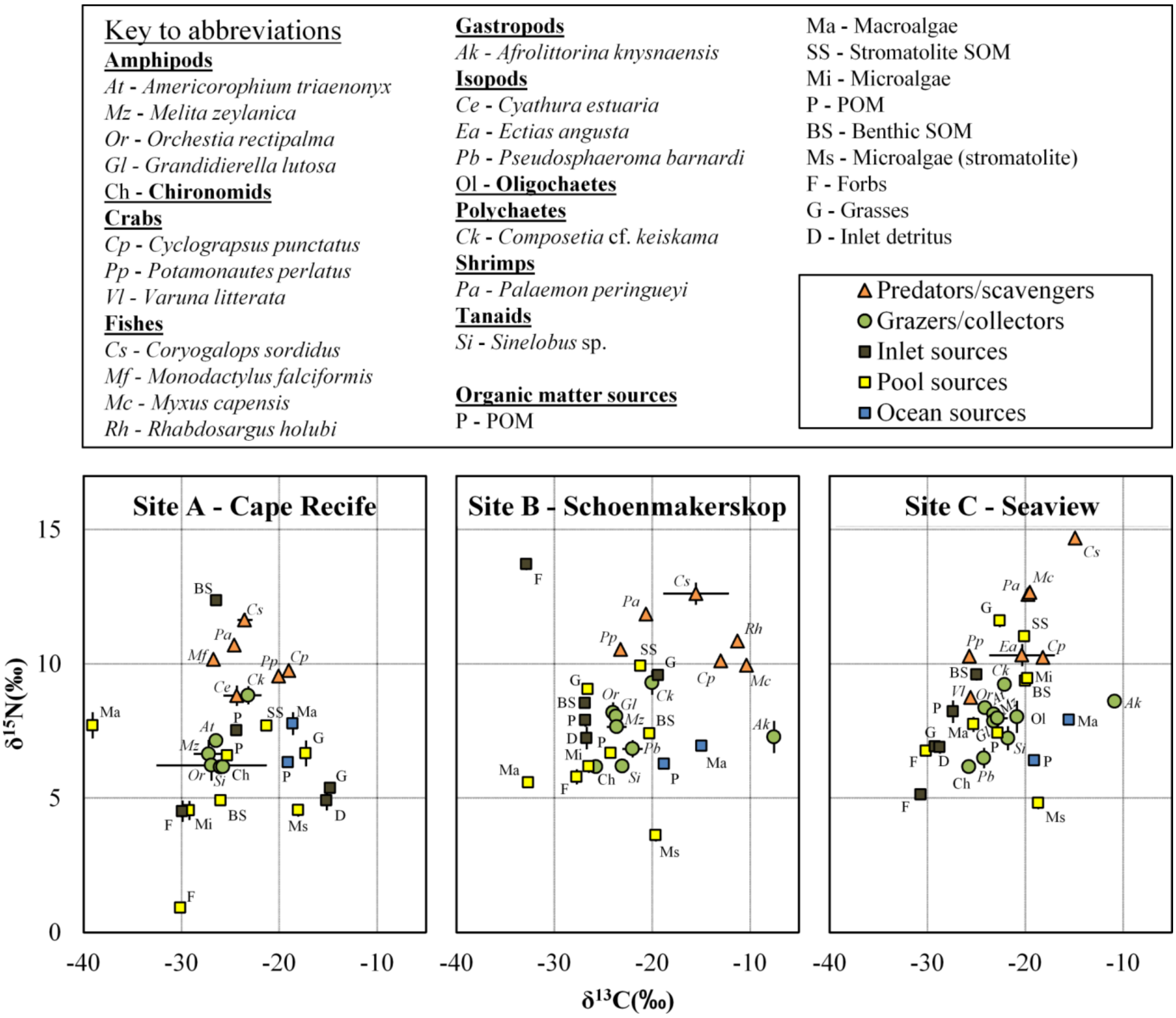

Figure S2. Stable isotope biplot of carbon (δ^{13}C) in relation to nitrogen (δ^{15}N) for trophic components from three living stromatolite locations along the South African coastline during summer 2016 (February). Infaunal and epifaunal samples are combined in this representation. Chironomid samples represent a single, multi-site sample combined prior to spectrophotometric analysis.

Table S2. Relative abundance (from Rishworth *et al.* [29]) within the stromatolite matrix of the main barrage pool as well as site-specific samples collected (‘X’: sufficient material from samples) of primary consumer metazoans from three sites along the South African coastline. W: winter 2015 (Rishworth *et al.* [28]); S: summer 2016 (this study).

			**Site A - Cape Recife**			**Site B - Schoenmakerskop**			**Site C - Seaview**		
**Class**	**Order**	**Species (or Family)**	Density (n cm^{-2} ±SD)[1]	W[2]	S[3]	Density (n cm^{-2} ±SD)[1]	W[2]	S[3]	Density (n cm^{-2} ±SD)[1]	W[2]	S[3]
Clittellata	Haplotaxida	Naididae, Enchtraeidae	0.6 ±1.1			5.4 ±6.7			3.2 ±3.2		X
Malacostraca	Amphipoda	*Orchestia rectipalma*	2.4 ±3.0	X	X	1.8 ±3.0	X	X	1.6 ±2.0	X	X
		Melita zeylanica	0.0 ±0.1	X	X	0.8 ±1.1	X	X	0.6 ±0.5	X	X
		Americorophium triaenonyx	0.0 ±0.0		X	0.1 ±0.3			0.0 ±0.1	X	X
		Grandidierella lutosa	0.0 ±0.0			0.0 ±0.0		X	0.0 ±0.0		X
	Tanaidacea	*Sinelobus* sp.	1.4 ±1.9	X	X	3.5 ±4.0	X	X	2.5 ±4.6	X	X
	Isopoda	*Pseudosphaeroma barnardi*	0.3 ±0.7			0.2 ±0.3	X	X	0.2 ±0.2	X	X
Polychaeta	Phyllodocida	*Composetia* cf. *keiskama*	0.7 ±0.8	X	X	1.8 ±1.6	X	X	1.6 ±1.3	X	X
Insecta	Diptera	Chironomidae	0.5 ±0.9	X	X	1.0 ±1.5	X	X	1.4 ±1.8	X	X
Gastropoda	Littorinomorpha	*Afrolittorina knysnaensis*	0.0 ±0.0			0.0 ±0.0	X	X	0.0 ±0.0		X

[1]Rishworth *et al.* [29]; [2]Rishworth *et al.* [28]; [3]This study

Table S3. Mean (±SD) $\delta^{13}C$ isotope signatures for organic matter sources associated with three living stromatolite locations along the South African coastline during February 2016 (summer). These are compared in relation to organic matter source grouping and sampling site using a generalised least squares (GLS) model which accounts for the source-specific variance (var). The directional coefficient (C) and test (t) significance (P) thereof are also shown.

	**$\delta^{13}C$**	*GLS model*			
	**Mean (±SD) ‰**	**C (±SE)**	**t**	**P**	**var**
Stromatolite microalgae	-18.8 (±0.8)	*			0.13
Inlet detritus	-23.5 (±7.3)	-4.7 (±4.5)	-1.1	0.31	1.00
Inlet POM	-26.2 (±1.6)	-7.4 (±1.4)	-5.4	< 0.001	0.28
Inlet SOM	-26.1 (±1.0)	-7.3 (±0.6)	-11.6	< 0.001	0.05
Ocean macroalgae	-16.4 (±2.0)	2.4 (±1.2)	2.1	0.05	0.23
Ocean POM	-19.0 (±0.2)	-0.2 (±0.7)	-0.3	0.79	0.10
Pool microalgae	-25.2 (±4.9)	-6.4 (±2.5)	-2.6	< 0.05	0.54
Pool POM	-24.1 (±1.3)	-5.3 (±0.7)	-7.6	< 0.001	0.09
Pool SOM	-22.1 (±3.4)	-3.3 (±1.8)	-1.8	0.09	0.39
Pool macroalgae	-32.3 (±6.9)	-13.6 (±3.7)	-3.7	< 0.01	0.81
Stromatolite SOM	-20.9 (±0.7)	-2.1 (±0.6)	-3.5	< 0.01	0.00
Cape Recife	-23.9 (±6.6)	*			
Schoenmakerskop	-23.5 (±5.0)	0.1 (±0.0)	>1000	< 0.001	
Seaview	-22.0 (±4.1)	1.2 (±0.0)	>1000	< 0.001	

*'Stromatolite microalgae' and 'Cape Recife' are the reference values for the GLS analysis

SOM (*sediment organic matter*); POM (*particulate organic matter*)

Table S4. Raw stable isotope values for all community components measured during summer 2016 at the three stromatolite locations. Winter data are presented in Rishworth et al. [28]. Relative position with the stromatolite matrix is represented as “Epi” (external) or “In” (within).

Site	Grouping/Species	Trophic level	Location	δ13C	δ15N	SD δ13C	SD δ15N	%C	%N
A	*Americorophium triaenonyx*	Grazer/collector	Epi	-26.4	7.1	0.0	0.0	33%	7%
A	Chironomid	Grazer/collector	Epi	-25.7	6.2	0.0	0.0	42%	10%
A	*Composetia keiskama*	Grazer/collector	Epi	-23.9	8.6	0.0	0.3	39%	11%
A	*Composetia keiskama*	Grazer/collector	In	-21.7	9.2	0.1	0.2	30%	7%
A	*Coryogalops sordidus*	Predator/scavenger	Epi	-23.5	11.6	0.8	0.3	41%	12%
A	*Cyathura estuaria*	Predator/scavenger	Epi	-25.1	8.7	0.2	0.3	42%	5%
A	*Cyathura estuaria*	Predator/scavenger	In	-22.9	9.1	0.0	0.0	35%	6%
A	*Cyclograpsus punctatus*	Predator/scavenger	Epi	-19.0	9.8	0.1	0.2	41%	11%
A	Inlet detritus	Terrestrial organic matter	Epi	-15.2	4.9	0.1	0.4	38%	1%
A	Inlet grasses	Terrestrial organic matter	Epi	-14.8	5.4	0.1	0.1	38%	2%
A	Inlet POM	Terrestrial organic matter	Epi	-24.4	7.5	0.2	0.0	10%	0%
A	Inlet SOM	Terrestrial organic matter	Epi	-26.4	12.4	0.0	0.0	10%	0%
A	Inlet succulents	Terrestrial organic matter	Epi	-29.9	4.5	0.1	0.4	40%	2%
A	Marine macroalgae	Ocean organic matter	Epi	-18.6	7.8	0.2	0.4	45%	3%
A	*Melita zeylanica*	Grazer/collector	Epi	-27.2	6.7	0.1	0.1	39%	7%
A	*Monodactylus falciformis*	Predator/scavenger	Epi	-26.7	10.2	0.2	0.1	40%	11%
A	Ocean POM	Ocean organic matter	Epi	-19.1	6.3	0.0	0.0	10%	0%
A	*Orchestia rectipalma*	Grazer/collector	Epi	-30.9	6.6	0.0	0.0	34%	8%
A	*Orchestia rectipalma*	Grazer/collector	In	-23.0	5.8	0.0	0.0	32%	7%
A	*Palaemon peringueyi*	Predator/scavenger	Epi	-24.6	10.7	0.0	0.0	43%	12%
A	Pool grasses	Pool organic matter	Epi	-17.3	6.7	0.2	0.5	38%	1%
A	Pool microalgae	Pool organic matter	Epi	-29.2	4.5	0.1	0.4	20%	0%
A	Pool POM	Pool organic matter	Epi	-25.3	6.6	0.1	0.1	10%	0%
A	Pool SOM	Pool organic matter	Epi	-26.0	4.9	0.0	0.0	10%	0%
A	Pool succulents	Pool organic matter	Epi	-30.1	0.9	0.2	0.1	36%	1%
A	*Potomonautes perlatus*	Predator/scavenger	Epi	-20.1	9.5	0.2	0.1	41%	11%
A	*Sinelobus*	Grazer/collector	Epi	-26.0	6.2	0.1	0.0	37%	7%
A	Stromatolite macroalgae	Pool organic matter	Epi	-39.1	7.7	0.2	0.5	37%	1%
A	Stromatolite microalgae	Pool organic matter	In	-18.1	4.6	0.1	0.2	18%	0%
A	Stromatolite SOM	Pool organic matter	In	-21.3	7.7	0.0	0.0	8%	0%
B	*Afrolittorina knysnaensis*	Grazer/collector	Epi	-7.6	7.3	0.4	0.6	14%	1%
B	Chironomid	Grazer/collector	Epi	-25.7	6.2	0.0	0.0	42%	10%
B	*Composetia keiskama*	Grazer/collector	Epi	-20.2	9.3	0.1	0.4	42%	12%
B	*Composetia keiskama*	Grazer/collector	In	-19.8	9.3	0.2	0.5	34%	9%
B	*Coryogalops sordidus*	Predator/scavenger	Epi	-15.5	12.6	3.3	0.4	43%	12%
B	*Cyclograpsus punctatus*	Predator/scavenger	Epi	-13.0	10.1	0.1	0.1	37%	10%
B	*Grandidierella lutosa*	Grazer/collector	In	-23.7	8.1	0.0	0.0	29%	6%
B	Inlet detritus	Terrestrial organic matter	Epi	-26.7	7.2	0.1	0.4	38%	2%
B	Inlet grasses	Terrestrial organic matter	Epi	-19.4	9.6	0.4	0.2	40%	2%

Site	Grouping/Species	Trophic level	Location	δ13C	δ15N	SD δ13C	SD δ15N	%C	%N
B	Inlet POM	Terrestrial organic matter	Epi	-26.8	7.9	0.0	0.2	10%	0%
B	Inlet SOM	Terrestrial organic matter	Epi	-26.9	8.5	0.0	0.0	10%	0%
B	Inlet succulents	Terrestrial organic matter	Epi	-32.9	13.7	0.1	0.2	39%	3%
B	Marine macroalgae	Ocean organic matter	Epi	-14.9	6.9	0.2	0.1	49%	3%
B	*Melita zeylanica*	Grazer/collector	Epi	-24.2	7.6	0.1	0.2	38%	6%
B	*Melita zeylanica*	Grazer/collector	In	-22.5	7.7	0.1	0.0	35%	7%
B	*Myxus capensis*	Predator/scavenger	Epi	-10.4	10.0	0.3	0.1	41%	11%
B	Ocean POM	Ocean organic matter	Epi	-18.8	6.3	0.1	0.1	10%	0%
B	*Orchestia rectipalma*	Grazer/collector	Epi	-24.3	8.3	0.0	0.0	41%	8%
B	*Orchestia rectipalma*	Grazer/collector	In	-23.4	7.9	0.0	0.0	30%	7%
B	*Palaemon peringueyi*	Predator/scavenger	Epi	-20.6	11.9	0.0	0.1	41%	11%
B	Pool grasses	Pool organic matter	Epi	-26.6	9.1	0.1	0.2	40%	1%
B	Pool microalgae	Pool organic matter	Epi	-26.5	6.2	0.0	0.2	28%	1%
B	Pool POM	Pool organic matter	Epi	-24.2	6.7	0.0	0.1	10%	0%
B	Pool SOM	Pool organic matter	Epi	-20.3	7.4	0.0	0.0	10%	0%
B	Pool succulents	Pool organic matter	Epi	-27.7	5.8	0.3	0.3	41%	1%
B	*Potomonautes perlatus*	Predator/scavenger	Epi	-23.2	10.5	0.1	0.1	41%	11%
B	*Pseudosphaeroma barnardi*	Grazer/collector	Epi	-22.0	7.0	0.1	0.2	26%	3%
B	*Pseudosphaeroma barnardi*	Grazer/collector	In	-22.1	6.5	0.2	0.3	29%	4%
B	*Rhabdosargus holubi*	Predator/scavenger	Epi	-11.3	10.9	0.1	0.1	40%	11%
B	*Sinelobus*	Grazer/collector	In	-23.0	6.2	0.0	0.0	30%	6%
B	Stromatolite macroalgae	Pool organic matter	Epi	-32.7	5.6	0.1	0.2	38%	2%
B	Stromatolite microalgae	Pool organic matter	In	-19.6	3.6	0.1	0.2	19%	0%
B	Stromatolite SOM	Pool organic matter	In	-21.2	9.9	0.0	0.0	11%	0%
C	*Afrolittorina knysnaensis*	Grazer/collector	Epi	-10.9	8.6	0.5	0.5	15%	1%
C	*Americorophium triaenonyx*	Grazer/collector	Epi	-23.2	8.1	0.0	0.0	28%	6%
C	Chironomid	Grazer/collector	Epi	-25.7	6.2	0.0	0.0	42%	10%
C	*Composetia keiskama*	Grazer/collector	In	-22.1	9.2	0.1	0.1	33%	9%
C	*Coryogalops sordidus*	Predator/scavenger	Epi	-14.9	14.7	1.6	1.9	38%	11%
C	*Cyclograpsus punctatus*	Predator/scavenger	Epi	-18.2	10.2	0.1	0.0	37%	10%
C	*Ectias angusta*	Predator/scavenger	Epi	-20.3	10.2	0.1	0.4	35%	5%
C	*Ectias angusta*	Predator/scavenger	In	-20.4	10.6	0.1	0.2	32%	5%
C	*Grandidierella lutosa*	Grazer/collector	Epi	-23.2	7.9	0.0	0.0	41%	10%
C	Inlet detritus	Terrestrial organic matter	Epi	-28.7	6.9	0.5	0.1	40%	1%
C	Inlet grasses	Terrestrial organic matter	Epi	-29.2	6.9	0.1	0.4	41%	1%
C	Inlet POM	Terrestrial organic matter	Epi	-27.4	8.2	0.2	0.3	10%	0%
C	Inlet SOM	Terrestrial organic matter	Epi	-25.0	9.6	0.0	0.0	10%	0%
C	Inlet succulents	Terrestrial organic matter	Epi	-30.7	5.1	0.3	0.2	40%	4%
C	Marine macroalgae	Ocean organic matter	Epi	-15.5	7.9	0.2	0.3	30%	2%
C	*Melita zeylanica*	Grazer/collector	Epi	-23.0	8.1	0.1	0.3	37%	7%
C	*Melita zeylanica*	Grazer/collector	In	-22.5	7.8	0.0	0.0	32%	7%
C	*Myxus capensis*	Predator/scavenger	Epi	-19.5	12.7	0.2	0.1	40%	11%
C	Ocean POM	Ocean organic matter	Epi	-19.1	6.4	0.0	0.1	10%	0%
C	Oligochaete	Grazer/collector	In	-20.8	8.0	0.0	0.0	35%	8%
C	*Orchestia rectipalma*	Grazer/collector	Epi	-24.4	8.5	0.1	0.1	39%	8%

Site	Grouping/Species	Trophic level	Location	δ13C	δ15N	SD δ13C	SD δ15N	%C	%N
C	*Orchestia rectipalma*	Grazer/collector	In	-23.4	8.2	0.0	0.0	48%	8%
C	*Palaemon peringueyi*	Predator/scavenger	Epi	-19.7	12.6	0.1	0.1	43%	12%
C	Pool grasses	Pool organic matter	Epi	-22.6	11.6	0.3	0.4	47%	2%
C	Pool microalgae	Pool organic matter	Epi	-19.8	9.5	0.1	0.3	20%	1%
C	Pool POM	Pool organic matter	Epi	-22.8	7.4	0.1	0.0	10%	0%
C	Pool SOM	Pool organic matter	Epi	-20.0	9.4	0.0	0.0	10%	0%
C	Pool succulents	Pool organic matter	Epi	-30.1	6.8	0.1	0.0	41%	2%
C	*Potomonautes perlatus*	Predator/scavenger	Epi	-25.7	10.3	0.1	0.0	38%	10%
C	*Pseudosphaeroma barnardi*	Grazer/collector	Epi	-23.1	6.5	0.0	0.0	47%	4%
C	*Pseudosphaeroma barnardi*	Grazer/collector	In	-26.4	6.5	0.1	0.2	37%	4%
C	*Sinelobus*	Grazer/collector	Epi	-20.5	8.1	0.0	0.0	32%	6%
C	*Sinelobus*	Grazer/collector	In	-23.1	6.4	0.1	0.1	44%	7%
C	Stromatolite macroalgae	Pool organic matter	Epi	-25.3	7.8	0.1	0.3	41%	2%
C	Stromatolite microalgae	Pool organic matter	In	-18.7	4.8	0.2	0.4	25%	0%
C	Stromatolite SOM	Pool organic matter	In	-20.1	11.0	0.0	0.0	4%	0%
C	*Varuna*	Predator/scavenger	Epi	-25.6	8.7	0.0	0.1	39%	11%
